# Affections of the Nipple and Breast, Incident to Early Lactation

**Published:** 1877-12

**Authors:** Edw. Warren Sawyer

**Affiliations:** Lecturer on Obstetrics and Diseases of Children; 116 Vincennes Ave.


### EXAMINER.

Vol. XXXV.—DECEMBER, 1877.—No. 6.

### Original Communications.

### AFFECTIONS OF THE NIPPLE AND BREAST INCIDENT TO EARLY LACTATION.

By Edw. Warren Sawyer, M. D.,

[Lecturer on Obstetrics and Diseases of Children.]

(*A Lecture delivered at Rush Medical College, Chicago.*)

Gentlemen,—I propose to occupy the hour in considering some of the common affections to which the breast of the woman is liable, during early lactation. I may say that the management of the breast at this time is one of your most important duties in the lying-in-room; a duty that belongs exclusively to yourselves, and which should never be assumed by the monthly nurse. On account of the obstinancy at times of these affections, their successful management becomes one of your most perplexing cares; while their effect upon the sensitive parturient woman is sometimes so grave as to occasion you the greatest solicitude. But I hope to show that the care of the breast during the puerperal month is suggested by a few simple principles, which I shall endeavor to impress upon you.

To facilitate the study, I will speak first of the affections of the nipples, and second of the affections of which the breast proper is the seat. While this division will assist us in the class-room, you are not to infer that the separation of these affections is closely observed in practice. On the contrary, you will learn that the lesion to which the gland itself is most liable, is usually associated with, and dependent upon the affections of the nipple; and that the first cannot be remedied easily till the diseased nipple is cared for.

Before enumerating the several varieties of "sore nipple," as they are aptly called, the causation of these affections may be spoken of in a general manner. It has been often observed that women who are nursing for the first time, and those who have not nursed for a long time, are the special subjects of lesions of the nipples. This is because the cuticle covering the nipples becomes thin and tender from long disuse, when it is protected beneath the clothing from the influence of the sun and air. Some have said that women of blonde complexion are more liable to trouble from affections of the nipples than others; but I have never observed a marked difference in this respect.

There is a condition, however, which is intimately concerned in provoking these lesions; viz., that of bodily filth, and this, too, often in those women whose exterior gives assurance of quite another condition. During the last days of pregnancy there usually oozes from the nipple a few drops of fatty liquid, the imperfectly formed colostrum. The skin being for a long time covered with this matter, alternately dry and macerated, becomes tender and irritated, and ready to inflame as soon as the child is put to the breast. Even before the child is born, contact with the clothing may be sufficient to excite this inflammation.

Another determining cause of lesions of the nipple, is the undersize of the organ. The smaller the nipple, the greater the liability of having trouble during the first days of nursing; because the nipple is mumbled for a long time by the infant, in its efforts to keep its hold upon the organ.

In this connection, I may very properly speak of the advantages of putting the child very early to the breast, especially if the woman has small, or retracted nipples. The nipple is larger and more prominent just after labor, and is much more readily seized by the child than at a later date. If the putting of the child to the breast be for a long time delayed, until the breast is distended with milk, the small nipple is encroached upon, and retracted by the swelling of the breast, and seems even smaller than before, while the efforts which the child makes to draw it out will be painful and, for a long time, futile. Besides this, there is another consideration. The child is born with an instinct which enables it to nurse at the very moment of its birth. I have seen this instinct nearly extinguished by the usual delay in calling it into exercise; and the child has been forced to learn to nurse after many painful efforts. Unless an unusual degree of exhaustion of the mother positively contra-indicates such a course, I apply the child to the breast before I leave the house, during the first hour *post partum*. Such a procedure, as you know, has the further advantage of maintaining the uterus in a state of tonic contraction, which is the greatest safeguard against hæmorrhage after delivery.

The chief exciting cause of lesions of the nipple, is the mechanical irritation to which the part is subjected during the early days of nursing, under one or more of the determining conditions, of which I have just spoken. Practically, this is the only exciting cause which operates at this time.

A notion is entertained by many that certain irritating states of the secretions of the child's mouth, operate chemically in causing sore nipples. The affection called thrush, which is a fungoid exudation occurring in patches upon the tongue and inside of the cheeks, has been specially condemned, as the cause of the lesions in question. But I have never observed a single confirmation of this notion. Many women nurse a child with thrush, and escape sore nipples. And many others are the victims of these affections, whose children are free from thrush.

The division of affections of the nipple into several distinct varieties, which has usually been made, is of importance only because of the different degrees of painfulness of these affections, and because of the several palliative measures that have been invoked for their relief. But so far as the causation is concerned, and the radical treatment, the classification is of little practical value. They may be enumerated in the following order:

*Erythema* is a congestion of the superficial structures of the skin; usually the entire covering of the nipple is attacked, and the areola is also sometimes involved. The part is swollen, red and excessively tender. It stands out constantly from the breast, and the slightest contact with the clothing occasions pain. Under appropriate treatment this simple congestion of the epidermis will subside. Or, inflammation occurring, an exudation takes place beneath the superficial layer of the skin constituting

*Eczema*, in which a variable number of small vesicles are found upon the areola or sides of the nipple. If the affected nipples are placed at rest, the contents of these vesicles become dry, and a little crust is formed which soon falls off, and the part is well. On the other hand, if nursing is persisted in, these vesicles are ruptured, and a little abraded patch remains, which soon becomes an ulcerating surface. Two or more vesicles may coalesce and become torn, thus forming a linear abraded surface, which is torn deeper by nursing, until the crack extends through the cuticle, and even deeper. This is especially liable to occur at the junction of the nipple with its areola.

The contents of the inflammatory vesicles just spoken of are usually clear, or but slightly cloudy. Sometimes, however, under very vigorous nursing, other little blebs appear upon the sides and apex of the nipple, which are filled with blood. These may also be ruptured, and their site be marked by an abraded or ulcerating surface. As the integument becomes toughened by nursing, these little ecchymoses fail to appear.

*Fissures* about the nipple will more frequently be brought to your notice than the lesions just named, because they are more painful, and yield less readily to the sometimes injurious expedient resorted to by the nurse. Lesions of this kind do not usually occur till after you have given your convalescent patient into the charge of the monthly nurse. The pain, incident to nursing from the tender nipple, has been heroically borne till this time, when the part becomes cracked, and the woman has no longer the hardihood to persist in suckling her infant. You can have no adequate idea of the amount of suffering that the woman endures under these circumstances, until you have been told, as I have, that the agony of giving birth to the child was less than that of nursing.

Besides the pain, there is marked constitutional disturbance in the case of the woman. The appetite fails; she becomes discouraged and demoralized. Her night is disturbed, because in her fitful sleep she dreams of the cries of the child, that warn her of the time of nursing; and the dread of this rises constantly before her. Moreover, I have known the puerperal woman to have an elevated temperature, of even 103° F., which had no other explanation than the fissure of the nipple.

I have said that the woman has no longer the hardihood to nurse her child. This fact is of the greatest significance, for it determines another, and much more grave affection of the breast itself, of which I shall speak hereafter.

The direction of fissures is usually transversely across the nipple, at any point from the base of the organ to its tip. A common site is where the nipple joins the breast; though a nipple may be cracked high up on one side, and near its base upon the opposite. The length and depth of such cracks are variable; from the merest chap, scarcely long enough to be seen, to a clean-cut fissure, which nearly, or quite encircles the nipple, and threatens to amputate the part.

Fissures of this kind are so easily recognized, that they need never be overlooked. Simply putting the integument upon the stretch, is sufficient to reveal their exact site. But there is another variety of fissure not so readily seen upon inspection of the part; I speak of a fissure located upon the summit of the nipple. Most observers do not describe this variety, which actually occurs with the greatest frequency.

If the summit of the normal nipple is carefully examined, it will be seen to be surmounted by a score or more of cone-like papillæ, separated by little furrows, or valleys. The openings of the milk-ducts may be seen at the bottom of these valleys. A certain degree of care is necessary in your examination, or a fissure of this variety will be overlooked. The woman experiences a smarting pain during, and after, nursing; and sometimes there is blood upon the lips of her infant; but the sensations of the woman do not enable her to locate the lesion. If your examination is limited to an inspection of the part, you, too, will fail to see the cause of complaint. Separate the papillæ with the end of your thumb and forefinger, and at the bottom of the little furrows, of which I have spoken, you will see the fissure. A strong light and the aid of a lens will assist you in finding the lesion. It may seem a slight affair, so far as its length is concerned; or it may be a long zig-zag crack, extending in and out between several papillæ. I have never seen this fissure extend to a greater depth than the thickness of the skin, though some writers have described it, in extreme cases, as cleaving the nipple nearly to its base.

The manner in which this particular fissure is produced is, I think, worthy of a word in passing. You know that a skein of from fifteen to twenty milk-ducts passes through the central portion of the nipple, to open upon its summit. This central portion is not capable of much elongation. But this cone is enveloped in a case of integument, with a layer of loose, fat tissue interposed, which is capable of a very considerable degree of elongation. Now, during nursing, the outside structure of the nipple is drawn outward, beyond the central portion of the organ; in much the same manner as a thimble is raised from the finger. But the comparison is not strictly true, for the integument is very firmly adherent to the summit of the nipple, and limits the drawing out of its envelope. Prolonged, or very forcible nursing, tears the integument upon the top of the nipple, and causes the fissure in question. I have noticed that the general direction of these fissures upon the summit of the nipple is upward, towards the median line of the woman. This fact is of interest, when we remember that this general direction coincides with the chink between the jaws of the infant, when he is held in the usual nursing position.

I have already alluded to the general effect of severe lesions of the nipple upon the susceptible parturient woman: how she may become demoralized by pain, and the want of sleep incident to nursing, and the dread of nursing; and how you may fail to understand the cause of the elevated temperature, until you have examined her nipples. It may seem to you that the effects are out of all keeping with the trivial lesion which you find upon the part. Permit me here to caution you against thinking that the woman has too little cause for complaint. You will, I hope, remember that it is a real martyrdom which the woman suffers, who heroically nurses her child under these circumstances.

I come now to speak of the treatment of these lesions. When you read the medical journals more extensively, you will be astonished at the number of expedients and drugs which are, from time to time, recommended as specifics for sore nipples. While this very multiplicity of means embarrasses the reader who wishes to know how to treat these affections, it shows that the subject is sufficiently practical to be constantly before the general practitioner. It is not worth your time for me to attempt to enumerate the many details of treatment that have been offered. Fortunately, it is only necessary for you to burden your memory with but a few very simple principles of treatment.

Practically the radical treatment of these various affections is the same for all. Knowing that the immediate cause of these lesions is the mechanical irritation of the nipple, by the mouth of the child, there arises from this fact one indication of paramount importance; viz., to remove the organ from this irritation. This is the maxim in treatment which I cannot too strongly emphasize. Now, how can this be carried out? You cannot interfere with the function of one breast without compromising the gland. Nor can you partially empty both breasts, for the same reason. To remove the nipple from the cause of injury, at the same time to secure the safety of the gland, is the great desideratum. To accomplish this, a variety of shields have been devised for the breast. They are all made to cover the nipple, as a thimble, and rest air-tight upon the breast, and are surmounted with a false nipple of caoutchouc. If aspiration is applied to the rubber nipple, an incomplete vacuum is formed in the shield, which is filled by the milk from the ducts. The most efficient, at the same time, the cheapest and most simple variety of nipple shields, is this plain bell-glass, with a broad base to rest upon the breast.[1] If the bearing surface of the shield is wetted with saliva or glycerine, it will fit upon the breast more tightly.

It is true that the use of this kind of cupping glass is not always free from pain, because it induces an engorgement of the nipple; but I know of no other way in which the nipple can be placed so nearly at rest, at the same time that lactation is kept up. The immediate relief and rapid improvement which have followed the temporary use of this means, in cases where all sorts of lotions and collodion coatings had failed, have often surprised me.

It sometimes happens that the child is not vigorous enough to draw the milk through the shield; then the aspiration should be made by an adult. In this manner the breast can be sufficiently emptied to protect it from trouble, until the lesion of the nipple has recovered. In the meantime the child can be sustained from the sound breast. Sometimes both nipples are the seat of lesions at the same time. Under these circumstances, if the child is unable to draw the milk through the shield, some other means must be devised, so that the milk may be saved for the infant. I now make use of that simple form of breast-pump, which is practically like the nipple-shield of which I have spoken. The bell-glass has a diverticulum, in which the milk is collected; and in place of the false nipple, there is a large rubber bulb, by means of which the vacuum is made. The milk can be poured from the receptacle, as often as filled, kept warm, and fed to the child with the spoon, or the simple nursing bottle.

This use of the breast-pump is the only use I ever have for it; I believe it to be its only proper use. If it were not neces-

1 Known as Maw's.

sary to collect the milk for the child, the simple emptying of the breast could be done much more safely through the nipple shield. In all cases, I believe it good practice to complete the emptying of the breast with the shield, after the pump has been used.

I have spoken at length of the means of protecting the nipple from the irritation of immediate nursing, because this is, by far, the most important indication of treatment. In the great majority of cases, nothing more than this is required to cure the lesion. Usually, however, there is associated with the ulcers and fissures of the nipple, an inflammation of the cuticle covering the part, which renders the organ excessively tender, and calls for palliative treatment. For this purpose you will find the use of the benzoated zinc ointment most grateful. Wash the part carefully with castile soap and water, and cover the entire nipple and areola with the ointment. It is not necessary to remove the ointment before drawing the milk through the shield. A few drops of carbolic acid, rubbed into the ointment seem to increase its efficiency.

For the very red, and highly inflamed nipple, I have found the solution of acetate of lead a very cooling and agreeable lotion. It is always necessary to wash the lead from the part before the milk is drawn, else, as has been said, the child will be injured by it.

At times the ulcers show an indolence which is easily corrected by touching them with lunar caustic. Ordinarily the ulcer needs only to be kept clean.

If the fissures are deep, and their edges are pulled apart by aspiration, occasioning great pain and a little hemorrhage, it is a useful expedient to glue the wound by coating it with collodion.

Holding as I do that, in the great proportion of cases of these affections, especially in primiparæ, the determining cause is in the fact that the nipple is undersized, I am led to speak of the means of correcting this condition before the end of pregnancy. You will not often have an opportunity of becoming familiar with the breast of the young woman before her confinement; but I have more than once, in the primiparous woman, seen a nipple which really did not project beyond the surface of the breast, but seemed rather withdrawn into the latter. Undoubtedly the usual habit of wearing corsets with breast pads is to be condemned as the cause of this flat nipple. If you are consulted, the entire condition is susceptible of change. I direct the woman, during the last months of pregnancy, to make traction upon the nipple with her fingers, at least twice daily. This method is much more effectual than the many varieties of air-pumps sold for the purpose. In fact, the use of these pumps should be interdicted, for they sometimes excite the breast to premature activity.

If you find this flat nipple for the first time after confinement, you may invoke the use of the old-fashioned vacuum to draw it out. A colorless glass bottle, holding a pint or more, with a mouth at least an inch wide, may be filled with hot water. After pouring out the water the bottle is to be inverted over the nipple. The atmosphere within the bottle becomes rarified, and a state of vacuity is approached as the bottle cools, which draws the enclosed part into its cavity. A new clay pipe, such as smokers use, is often used for the same purpose. When once the nipple is large enough for the infant to seize, it will be kept projecting by the efforts at nursing.

Associated with the small nipple, and in many others also, there is a degree of tenderness which is, as I have said, a cause of subsequent trouble. The long continued contact of alcohol does harden the cuticle, and render it less sensitive. Begin early in pregnancy to bathe the nipples and areolæ twice daily with bay-rum, cologne water, or diluted alcohol. The well-known domestic solution of borax, to which alcohol is added, I have found useful for this purpose.

The affection of the breast that you will frequently meet with in the primiparous woman, and exceptionally in others also, is an inflammation of the organ. Perhaps more has been written upon this than upon any other lesion, unless we except the so-called puerperal fever. It must be admitted that the subject is worthy of the attention it has received, because of the gravity of the general and local effects of the affection, and because an inflamed breast is often regarded as a reproach to the attendant. While it is true, a breast may inflame and suppurate under the most skillful management, it cannot be denied that this result more often occurs under circumstances entirely preventable.

Inflammation about the breast is often thought to be connected exclusively with the puerperal state, or the function of lactation. This is an error. I have recently seen an abscess of the breast in a woman who had nursed her infant for more than a year. Moreover, a form of abscess is met with in new-born children, of both sexes ; and mammary abscess has been reported as occurring in adult males. These facts are sufficient to show that the breast may inflame independently of the special conditions of the newly delivered woman. We shall see presently, however, that exceptional inflammations of this class, have quite another significance than the usual cases occurring during convalescence from confinement.

But the inflammations of the breast of the lying-in woman are not always of similar causation and course. This fact leads to the division of mammary inflammation into two varieties, which I will describe, after calling to your mind some facts concerning the anatomical structure of the breast.

Each breast, as you know, is made up of from twelve to fifteen distinct, wedge-shaped lobes, arranged circularly in the breast ; the small end of the lobes looking toward the centre of the circle. These lobes, in their turn, are made up of many smaller parts, called lobules. Each lobule has an excretory duct, which joins the duct from the neighboring lobule ; the whole uniting to form one common duct for that lobe. These grand canals from the different lobes, called the lactiferous ducts, converge toward the centre of the breast, where they turn upward and pass through the nipple, to open upon its summit, as has been already described. The lactiferous ducts do not anastomose with each other, but remain distinct throughout. Just beneath the areola, each duct suddenly dilates into a much larger canal, only to resume a still smaller calibre an inch nearer its opening. These dilatations are called milk-sinuses, and serve as reservoirs for the milk. Each lobe is a distinct gland, of the compound racemose variety, and may be looked upon as a miniature breast, capable of executing its function independently of its neighbor. In fact, it does just this, when a part of the breast has been destroyed by disease.

But there is another tissue which enters largely into the formation of the breast, and which, so far as the present subject is concerned, is scarcely second in importance to the glandular structure just described. The different lobes of the breast are bound together by a quantity of intervening loose adipose tissue, which also covers the breast subcutaneously, everywhere upon its round aspect, except beneath the areola. It is the deficiency of the fat tissue at this part, that gives this disk a different hue from the surrounding covering. The breast itself rests upon a bed of loose cellular tissue, which attaches the organ to the fascia of the larger pectoral muscle.

From this it is seen that the gland tissue proper is everywhere enveloped in this loose cellular tissue, which sends down partitions between the smallest lobules. The great bulk of the breast is made up of this adipose tissue, which is so variable in quantity in different individuals, that one may have a large, and another a small breast. It is probable that the gland tissue varies but little in different breasts.

The fact of this double structure of the breast establishes a natural division in the inflammations which attack the organ, according as the glandular or the cellular structure is the point of departure. These varieties require separate consideration. I will speak first of that variety in which the glandular element is the starting point, known properly as—

*Mastitis.* The circumstances attending the establishing of lactation are such as determine an unusual amount of blood to the breast. This hyperæmia is shown in the increase in size of the organ. It is also harder to the touch, and so much heavier that it seems to drag away from its attachments as the woman lies in bed. There is a sensation of fullness experienced, at times amounting to positive pain. This local condition is sufficient to slightly elevate the temperature, and to accelerate the pulse. The term milk-fever is applied to this group of phenomena, about which the older teachers have written so much; but whose very existence even, is denied by many modern writers. When you come to practice, and to observe everything about your lying-in patient as you should, you will be able to judge for yourselves, whether milk-fever is a real specific condition, or a chimera of the ancients. For my part, I believe it does exist. But I must warn you that milk-fever, at most, is only a slight, and transitory elevation of the temperature, not preceded by a marked rigor; and that any unusual departure from this rule, indicates some traumatism about the parturient canal, or phlegmasia within the pelvis.

It has been said that the great functional activity of the breast is sufficient to determine a true inflammation within the organ. I believe this conveys a most erroneous idea as to the causation of mammary inflammation. In no instance in the economy, is there such a lack of harmony between the end to be attained and the means by which it is attained. Under no circumstances will the physiological hyperæmia of a gland, end in suppuration, if left to itself. You will remember that the various glands within the body, of which the breast may be taken as the type, have a double function; viz., to furnish their peculiar secretion and to empty themselves through their ducts. It is only when the harmony between these two functions is disturbed, that inflammation is induced in the gland.

Now, with reference to the breast, it is an important fact that you may suddenly cease drawing milk from the organ at any time, or refrain from drawing the first secretion after confinement, and if the breast is left absolutely alone, it will not inflame. On the other hand, if the breast is partially emptied to-day, and a little less milk is drawn to-morrow, in the hope that the function of lactation will be gradually extinguished, the breast will almost positively inflame. The maxim is: it is safe to draw no milk from the breast, but it is extremely hazardous to incompletely empty the organ.

Let us examine the operation of this law. If the organ is left quite to itself, withholding all stimulants to the breast and nipple, I mean by this all stimulating friction and all efforts at aspiration through the nipple, a small quantity of milk will escape spontaneously, probably enough to empty the reservoirs. The hyperæmia of the gland will subside, secretion will be rapidly suppressed, and the gland will gradually return to the condition of the organ before pregnancy. In a word, nature will observe the harmony between secretion and excretion, which is always safe for the secreting gland.

Let us suppose the opposite course to be followed, and the gland to be stimulated. By stimulating the gland I mean, first, the use of all means intended to draw the milk, either by immediate nursing (the infant or a pup) or by mediate nursing, (the breast-pump or shield); second, all frictions applied to the breast, or stimulating embrocations. All these measures cause the gland to furnish more milk than can be carried off. In a word, the harmony between secretion and excretion is interfered with.

These facts are strikingly confirmed in the lower animals. Who ever saw an abscess in the milk gland of the bitch, or cat? And yet the young of these animals are often taken from them without a thought! My teacher used to tell his medical class of a tender-hearted spinster whose cat lost her kittens upon the third day. The mistress, having a vague idea that her pet must suffer from distended mammæ, milked the cat for three days. The result was a mammary abscess—the only one of the kind on record. The domestic cow is not infrequently the victim of mammary abscess, when her calf is taken from her; but this is because she is milked. The buffalo cow, on the other hand, often loses her calf, but she is never milked, and hunters have told me that she never has mammary abscess.

To return to the woman. You have seen that nature has dictated the course to be followed. The danger to the breast is when the attendant follows an opposite course, and stimulates the breast to secrete more milk than can be drained away, because of some obstruction at the outlet of the milk canals. This leads to the inquiry, what is the nature of this obstruction? In other words, what is the real exciting cause which usually operates in obstructing the breast of the recently delivered woman?

I can have no doubt that the cause above all others is to be looked for in the graver lesions of the nipples, which have been described. The effect of these lesions is three-fold: First, the act of suckling gives the woman such pain that she is led to neglect her breast. Second, the inflammation of the epidermis of the nipple may extend into the duct itself, and thus occlude the latter. Third, the excessive irritation, of which the exterior of the nipple is the seat, may excite a spasmodic closure of the milk-ducts as they pass through the nipple; for in this part they are surrounded with muscular fibre. These latter complications are demonstrated in those cases where the woman heroically endures the agony of suckling, and yet the most forcible efforts at aspiration fail to draw milk from the breast.

Dr. M. O. Jones, of this city, narrated to me the instance of a woman under his charge, whose breast threatened to suppurate in one of its lobes. He was of the opinion that the affected part had not been completely emptied. After finding upon the top of the nipple an orifice corresponding nearly to the affected lobe, he passed a small probe, deep into the duct, which overcame the obstruction. Subsequent nursing relieved the breast, and the threatened abcess immediately disappeared. Perhaps with the aid of a lens to find the orifice, this quite original procedure of Dr. Jones, might be often used in practice.

You will hear much, in the lying-in room, of milk becoming curdled in the breast, so that it cannot be drawn. There is as little ground for this, as for many other phantoms pointed out by the nurse. I have often seen, when very forcible aspiration has been made through the nipple shield, little plugs of thickened milk drawn out, and have been led to ask if the milk, which is allowed to remain for a time in the reservoirs, does not become so changed as to obstruct the milk-ducts.

Besides this chief cause of glandular inflammation, you are not to forget that many other accidental causes, as for instance a blow upon the active breast, may operate to excite inflammation of the organ.

I have spoken at considerable length of the manner in which glandular inflammation is set up, because if this part of the history of the affection is well understood, you can do much to arrest the calamity; and this is to be regarded a greater triumph of art than the skillful management of a breast already containing an abscess.

The symptoms of mastitis may be briefly enumerated. They are those of circumscribed inflammation elsewhere. Pain is present with the first evidences of congestion, and increases in severity, till the inflammation has culminated in suppuration, and the pus has been evacuated. It is described at first as an aggravation of that sensation of engorgement of the breast, which the woman has experienced before. It soon becomes sharp, and cutting, extending toward the shoulder, and down the arm. The pain of a highly inflamed gland is truly excruciating, depriving the woman of sleep, and soon exhausting her. In addition, there is a degree of tenderness of the part, which makes the slightest pressure unbearable, and limits your examination to the most careful handling of the organ.

Heat and redness are the concomitants of inflammation within the breast as elsewhere. The woman sometimes describes her pain as of a burning character. A surface thermometer applied to the breast would show the temperature of the part, elevated above the general temperature of the body.

Redness is not apparent in early inflammation of the gland. In fact a lobe within the breast may be inflamed for days, before any cutaneous redness is seen. It is only when the cellular tissue is involved with the glandular, that the inflammation is superficial enough to redden the skin. The absence of cutaneous redness is one of the features which distinguishes true mastitis from superficial cellulitis.

Swelling of the breast is noticed in the earliest inflammation of the gland. But the breast also is greatly enlarged when it is only engorged to a physiological extent. Very often, in the case of the lying-in woman, you will hear of "the breasts beginning to cake," and of a threatened "bealing" in the organ. This occasions the nurse much solicitude,

She means that there is a circumscribed tumor within the breast, which she fears will end in suppuration. Now, these cakes are of common occurrence throughout the course of lactation. They should never, however, persist after a good nursing. If the cake does not disappear after the breast is emptied, it indicates the beginning of inflammation.

The swelling not only makes the individual lobe more distinct and enlarged, but the entire breast protrudes more—in an unsymmetrical manner—when but one lobe is the seat of the inflammation. The skin over the affected part becomes tense, shiny and white. With mastitis there is the usual sympathetic swelling and tenderness of the lymphatic ganglia within the axilla.

Beside the foregoing local effects of mastitis, the woman experiences the general disturbances of inflammation. Shudderings often occur during the establishment of lactation. These persist, and become more frequent as the physiological engorgement shades into incipient inflammation. The temperature is elevated, and the pulse accelerated. The woman has flushes of heat, and sweats profusely at night. A well marked rigor usually ushers in the formation of pus.

The *ensemble* of these phenomena is such as to seriously interrupt convalescence in the puerperal woman, and to impress the attendant with the gravity of the affection.

*Cellulitis.* The second variety of inflammation of the breast is an inflammation of the cellular tissue, which everywhere pervades the organ. That cellulitis may occur independently of glandular inflammation, is shown by those instances of furuncular inflammation in the breasts of women who have long ceased nursing, many months after confinement, in infants of both sexes and in men. But puerperal cellulitis may occur independently of glandular inflammation, as I have more than once verified.

Beside the general determining causes of furuncular inflammation that operate with some subjects, as for instance, those with scrofulous dyscrasia, there are many special causes of mammary cellulitis. A blow received upon the breast, even a long time before lactation may be sufficient to light up an inflammation when the breast is in a state of great activity. Writers speak of a miasmatic cause of this affection, which operates one year and not the next. But the exciting cause which operates in the great majority of cases, is an inflammation of the gland tissue proper. Under these circumstances mammary cellulitis is to be regarded as periglandular inflammation. In this connection I may state an observation suggested by a case. The gland tissue may be the starting point of inflammation which subsides after the process has been communicated to the cellular tissue and only the latter suppurate, the lobe escaping.

Now, any portion of the cellular tissue of the breast may be the seat of inflammation. This has given rise to the three distinct varieties of cellular abcess described by writers; the subcutaneous, glandular, and subglandular. Practically, it is of little importance whether the cellular tissue immediately beneath the skin, or an inch deeper in the breast, is inflamed, provided the abscess points externally. But that form in which the pus is pent up between the gland and the pectoral muscle, has a greater claim to be considered separately.

The symptoms of phlegmonous inflammation in the anterior half of the breast, are those of glandular inflammation of the organ, though less severe. Pain especially is much less noticeable. I know a lady who had six phlegmons in one breast, and four in the other, who scarcely complained of pain. A slight rigor would give warning of the successive abscesses. In this case the function of the breast was not apparently disturbed.

Unless the inflammation is deep in the breast, the skin over the inflamed part very early looks bluish-red. The swelling of the organ is not great, except in case of multiple abscesses. As a rule, cellulitis of the breast runs its course much more rapidly, and with less general and local disturbance, than mastitis.

In that form of mammary cellulitis called sub-glandular, the inflammation may have originated in the bed of loose tissue upon which the gland rests; or it may have extended from some point of the inter-lobar tissue, deep within the breast.

The early recognition of this variety is attended with much difficulty. The recurring rigor, or constant chilliness, indicates inflammation, which the pain locates within the breast. But there is no redness of the skin, and if the organ is palpated from side to side, no great pain is caused. But press the organ down hard upon its bed, and a sharp, cutting pain is elicited, quite characteristic of the lesion. Soon the breast is protruded by the engorged vessels at its base and the accumulation of pus, and the case is no longer obscure. The organ may be increased to three times its natural size. The swelling is not confined to the breast. I have seen the front and side of the thorax, and the corresponding shoulder and arm, extremely œdematous in a case of sub-mammary abscess. When a sub-glandular abscess has pointed, usually upon the lower margin of the breast, the skin becomes bluish-red; but until this time the covering has been uniformly white and waxy.

I have deferred speaking of the treatment of mammary inflammation till this time, because certain principles of treatment apply to both glandular inflammation and cellulitis.

An important enquiry is at once suggested: Will the inflammation amount to suppuration? If it is of the cellular variety, and accompanied with enough disturbance to place the existence of a phlegmon beyond question, especially if there is a circumscribed redness of the skin, I believe that suppuration is inevitable, even if it has not already taken place; and time should not be expended upon measures called antiphlogistic. On the other hand, a lobe of the glandular tissue may be hard, well defined and present evidences of incipient inflammation, and still not suppurate.

If either variety is recognized early, put the patient upon a low diet, and keep the bowels moderately relaxed, unless some more important puerperal condition precludes such a course. Much can be done locally to palliate, and perhaps to cut short the inflammation. Ice, or ice-water is often agreeable, and I have seen it effectual. Remember, however, that if you make cold applications to an inflamed breast, great caution must be observed. If the cold is withdrawn too soon, the severity of the inflammation is increased by the reaction that follows, and the end of that case is worse than the beginning. The following evaporating lotion may be used, however, without restrictions, and I have found its effect cooling and grateful: Dissolve fifteen grains of muriate of ammonia in an ounce each of dilute acetic acid, alcohol and water. Two thicknesses of muslin are to be wetted with this lotion, and kept applied to the part.

In inflammation of the gland itself, certain measures have been much extolled, because of their supposed effect in resolving the inflammation; viz., friction over the inflamed lobe, and the use of drugs. Of the first, I can say that I have rubbed the breast after the most approved fashion, and while I have imperfectly emptied the breast by this means, I am convinced that the inflammation was aggravated. Rubbing stimulates the gland to increased activity, and if there is any obstruction to the flow of milk, you have increased the danger.

The drug named phytolacca decandra, which is the "garget-root" that farmers give to cows under like circumstances, has been recently much employed in arresting glandular inflammation. In the single instance in which I know of its being tried, the process of inflammation was unusually slow, still it ended in an abscess. In giving such medicines you are not to forget that they usually disturb digestion, which fact more than offsets the uncertain effects of fallacious specifics.

The end which you are to have in view, is keeping the breast safely empty. Under most circumstances you can accomplish this by putting the child early and frequently to the breast; or, if the efforts of the child are too feeble, invoke the aid of an adult, through the shield if mediate suction is precluded. By this plan you will often be gratified to see a breast become soft that had been given over to suppuration.

If the lesions of the nipple constitute an obstacle not overcome by these means, the case is most unfortunate, and I think quite exceptional. The attendant is in a dilemma. Upon the one hand is the almost certain occurrence of abscess, because the gland is distended, and, upon the other, the lesions of the nipple which prevent nursing. Dr. Bedford has aptly called this being placed between Scylla and Charybdis. If such a case is presented to me, where the child and the adult fail to draw milk through the closed ducts, I would not hesitate to etherize the woman, which will relax the spasm of the ducts, and make powerful suction through the nipple. Failing in this, I should, with a lens, seek the orifice of the ducts involved, and pass into them a lachrymal probe, as Dr. Jones did.

In conjunction with these measures, I have for four years been accustomed to apply a binder very tightly about the thorax, wide enough to include the whole of both breasts, sometimes putting a sponge over the inflamed part, under the binder. This serves as a sling, which supports the heavy, tender breast, and, by compressing it against the ribs, lessens the engorgement of the organ. The bandage may be removed long enough to permit nursing from the breast, which is better than letting the nipple through an aperture in the binder.

In this connection I may say that when the child is weaned, or when the child is born dead, I never use any other measure to prevent mammary inflammation, and have never seen an abscess under these circumstances.

The treatment of mammary inflammation when suppuration is inevitable, may be summed up in a few words. You may know that the abscess has begun to form, when all the symptoms indicate increasing severity of the process: recurring rigors, rising temperature, and increased pain. Under these circumstances you will aim to make the suppuration as rapid as possible. Cover the breast with a thick, hot poultice, of flaxseed meal, spread upon oiled silk, or other impervious material, and change every third hour. A drachm of laudanum may be poured on that part of the poultice covering the abscess.

As soon as the pus has become subcutaneous it should be evacuated. Here let me caution you against making uncertain incisions into the breast. A good rule to follow is, wait until a circumscribed redness of the skin indicates the focus of the abscess. If your finger is passed over the deepest colored part of the cutaneous blush, it will reveal a crater just beneath the integument. At this point you may safely evacuate the pus, using a stiff-handled bistoury for the purpose, and plunging the blade to the depth of half an inch. It is desirable that no part of the areola should be included in your incision. It may be that your incision has opened a branch of the milk-duct, and milk flows out with pus. This is especially liable to happen if the abscess is near the areola; and this has been called a milk abscess by some writers.

Concerning the future of the breast the seat of abscess, it is well for you to know that the lobe which has suppurated will probably never again perform its functions.

In case of sub-glandular abscess, it is a triumph if you have early detected the presence of pus. An exploring needle passed into the tissue beneath the gland will aid you in this. If the exploration reveals pus, pass a probe or director into the track of the needle which will guide the knife. If this abscess is long neglected, the pus may burrow downward, and point far below the breast. I have attempted the evacuation of mammary abscess with the pneumatic aspirator, with the most unsatisfactory result.

I trust that I need hardly indicate to you that the subsequent management of each individual case will be suggested by your knowledge of those principles of surgery, in which you are so well instructed.

116 Vincennes Ave., Nov., 1877.

